# β-alanine alleviates gout by inhibiting NLRP3 inflammasome activation

**DOI:** 10.1093/jmcb/mjag004

**Published:** 2026-04-01

**Authors:** Xia-Ying Chen, Peng-Peng Zhu, Ying Yuan, Min-Yi Feng, Yu-Qin Xu, Liu-Bing Yu, Wen Xue, Yu Yu, Tao Li, Tao Zhou

**Affiliations:** Institute of Translational Medicine, Zhejiang University, Hangzhou 310029, China; Nanhu Laboratory, National Center of Biomedical Analysis, Beijing 100039, China; Institute of Translational Medicine, Zhejiang University, Hangzhou 310029, China; Nanhu Laboratory, National Center of Biomedical Analysis, Beijing 100039, China; Institute of Translational Medicine, Zhejiang University, Hangzhou 310029, China; Nanhu Laboratory, National Center of Biomedical Analysis, Beijing 100039, China; Nanhu Laboratory, National Center of Biomedical Analysis, Beijing 100039, China; Institute of Translational Medicine, Zhejiang University, Hangzhou 310029, China; Nanhu Laboratory, National Center of Biomedical Analysis, Beijing 100039, China; Nanhu Laboratory, National Center of Biomedical Analysis, Beijing 100039, China; Nanhu Laboratory, National Center of Biomedical Analysis, Beijing 100039, China; Nanhu Laboratory, National Center of Biomedical Analysis, Beijing 100039, China; Institute of Translational Medicine, Zhejiang University, Hangzhou 310029, China; Nanhu Laboratory, National Center of Biomedical Analysis, Beijing 100039, China; Institute of Translational Medicine, Zhejiang University, Hangzhou 310029, China; Nanhu Laboratory, National Center of Biomedical Analysis, Beijing 100039, China

**Keywords:** β-alanine, NLRP3, inflammasome, monosodium urate, gout

## Abstract

The NLRP3 inflammasome plays a pivotal role in mediating pro-inflammatory cytokine release and inducing pyroptosis. Its aberrant activation is implicated in various inflammatory diseases, including gout, a condition characterized by monosodium urate crystal deposition in the ankle joint. Here, we identify β-alanine, an endogenous amino acid, as a novel NLRP3 inflammasome inhibitor with promising therapeutic potential for gout. Mechanistic investigations reveal that β-alanine binds to NLRP3, sequestering it within the *trans*-Golgi network. This interaction disrupts NLRP3 inflammasome assembly, thereby inhibiting the secretion of interleukin-1β (IL-1β) and IL-18. Moreover, *in vivo* experiments demonstrate that β-alanine administration significantly alleviates monosodium urate crystal-induced inflammation and joint swelling in mice without evident toxicity. Collectively, our findings not only uncover a novel endogenous regulatory mechanism for NLRP3-driven inflammation but also position β-alanine as a potential therapeutic candidate for gout.

## Introduction

Inflammasomes are multiprotein signaling complexes that function as critical sentinels of innate immunity, detecting pathogen-associated molecular patterns (PAMPs) and damage-associated molecular patterns (DAMPs) to initiate inflammatory responses against microbial infections and cellular stress (Ratajczak et al., 2019; Vora et al., 2021). Among these, the NOD-, LRR-, and pyrin domain-containing protein 3 (NLRP3) inflammasome exhibits a broad range of pattern recognition capabilities, identifying both PAMPs, including bacterial toxins and lipopolysaccharide (LPS), and DAMPs, such as monosodium urate (MSU) crystals and adenosine triphosphate (ATP) (Fenini et al., 2017; Vande Walle et al., 2019). This versatility positions NLRP3 as a central hub for innate immune surveillance.

The NLRP3 inflammasome, comprising the sensor NLRP3, the adaptor apoptosis-associated speck-like protein containing a CARD (ASC), and the effector caspase-1 (Broz and Dixit, 2016), is activated through two sequential steps, termed as priming (Signal 1) and activation (Signal 2). The priming step is induced by stimuli such as LPS, which engages Toll-like receptors to induce NF-κB-dependent transcription of NLRP3 and pro-interleukin-1β (pro-IL-1β), followed by the activation step where PAMPs or DAMPs trigger NLRP3 inflammasome assembly (Broz and Dixit, 2016). Upon activation, NLRP3 undergoes a conformational shift from an autoinhibited state to an open conformation, enabling its trafficking from the *trans*-Golgi network (TGN) to the microtubule-organizing center (MTOC) via TGN dispersion. At the MTOC, NLRP3 interacts with NIMA-related kinase 7 (NEK7) and ASC to form the inflammasome complex, which activates caspase-1 (Chen and Chen, 2018; Magupalli et al., 2020; Arumugam et al., 2022; Schmacke et al., 2022). Active caspase-1 subsequently processes pro-IL-1β into mature cytokines and cleaves gasdermin D (GSDMD) (Fu and Wu, 2023), thereby triggering cytokine release and pyroptosis, a lytic programmed cell death critical for antimicrobial defense (Sharma and Kanneganti, 2021).

Despite its protective role, dysregulated NLRP3 activation drives pathogenesis in inflammatory diseases, including sepsis, atherosclerosis, and gout (Mangan et al., 2018; Xu and Núñez, 2023). Gout, for instance, results from the deposition of MSU crystals in articular and periarticular tissues (Dalbeth et al., 2019, 2021). These crystals function as DAMPs, triggering the activation of the NLRP3 inflammasome (Martinon et al., 2006). Chronic exposure to MSU crystal results in excessive inflammatory responses and progressive joint damage (Dalbeth et al., 2021). Such pathological conditions highlight the need for identifying novel regulatory mechanisms of NLRP3 inflammasome activation to develop effective strategies against NLRP3-related inflammatory diseases.

Emerging evidence reveals an intricate interplay between metabolic homeostasis and NLRP3 inflammasome activity (Ralston et al., 2017; Karasawa et al., 2018; Sharma and Kanneganti, 2021; Ortega et al., 2023). Metabolic perturbations, particularly amino acid dysregulation, have been implicated in NLRP3-associated inflammatory pathologies (Fu et al., 2022; Wang et al., 2024; Zhang et al., 2024; McManus et al., 2025), and altered amino acid networks are linked to gout risk (Mahbub et al., 2017; Joshi et al., 2023; Wang et al., 2023b; Han et al., 2025). In this context, non-proteinogenic amino acids, which are not incorporated into proteins but often serve important signaling or metabolic roles, have emerged as potential immunomodulators (Jakaria et al., 2019; Guerra et al., 2020; Koh et al., 2023). Among them, β-alanine is notable for its multifaceted physiological roles. It is derived from endogenous uracil metabolism and dietary sources and serves as the rate-limiting precursor for carnosine (β-alanyl-L-histidine), a dipeptide highly concentrated in the skeletal muscle and the central nervous system (Boldyrev et al., 2013). Carnosine functions as an intracellular pH buffer and antioxidant, supporting the established role of β-alanine in skeletal muscle function (Guerra et al., 2020). Beyond this, β-alanine shares structural homology with the neurotransmitters such as γ-aminobutyric acid, suggesting potential neuromodulatory functions (Tiedje et al., 2010; Zhang et al., 2021). However, the role for β-alanine in regulating innate immune responses remains largely unexplored.

Here, through the analysis of gout-associated metabolomics datasets, we identified β-alanine as a potent inhibitor of the NLRP3 inflammasome. Mechanistically, β-alanine suppresses the subcellular translocation of NLRP3 and subsequent inflammasome activation. Notably, β-alanine administration significantly mitigates inflammation in both LPS-induced sepsis model and MSU-induced gout arthritis model. Thus, our study suggests β-alanine as a novel metabolic regulator of NLRP3-driven inflammation, offering therapeutic potential for gout and other NLRP3-related inflammatory disorders.

## Results

### Identification of β-alanine as a NLRP3 inflammasome inhibitor from gout-associated metabolomic analyses

To identify potential metabolic regulators of the NLRP3 inflammasome relevant to gout pathogenesis, we analyzed public metabolomics datasets characterizing metabolic alterations in NLRP3-driven inflammatory conditions. A large-scale cohort-based metabolomics study revealed distinct plasma metabolite signatures associated with hospitalized incident gout, including glycine, glutamine, branched-chain amino acids (BCAAs), alanine, and histidine (Figure 1A; McCormick et al., 2024). Additionally, Kyoto Encyclopedia of Genes and Genomes (KEGG) pathway enrichment analysis of metabolomic data from MSU-stimulated cells (Renaudin et al., 2020) identified notable alterations in amino acid metabolism, particularly in the pathways of alanine, valine, β-alanine, glycine, histidine, and arginine (Figure 1B). Collectively, these analyses consistently highlighted dysregulated amino acid pathways associated with gout, many of which belong to the neutral aliphatic amino acid family, such as glycine, alanine, β-alanine, and BCAAs. Given the established role of glycine in regulating pyroptotic membrane integrity (Kayagaki et al., 2021; Borges et al., 2022) and the documented functional similarities between glycine and β-alanine as small neutral amino acids in certain biological contexts (Tiedje et al., 2010), we hypothesized that this subgroup of metabolites may modulate NLRP3 inflammasome activation.

**Figure 1:**
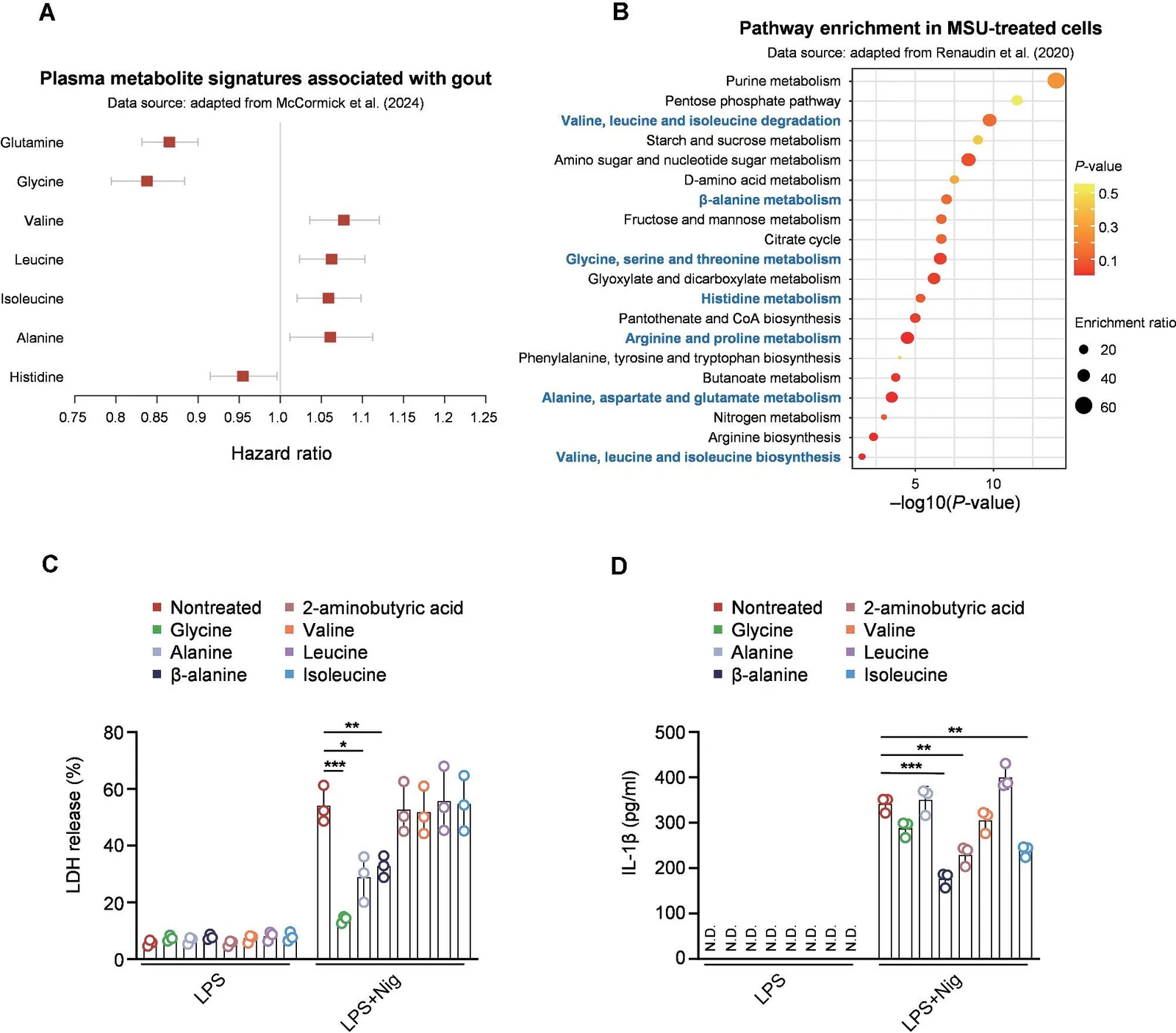
Identification of β-alanine as a candidate NLRP3 inflammasome inhibitor from gout-associated metabolomic analyses. (**A**) Forest plot of hazard ratio for plasma metabolite signatures associated with gout. (**B**) KEGG pathway enrichment analysis of metabolomic data from MSU-treated THP-1 cells. (**C** and **D**) iBMDMs were pretreated with 4 mM of the indicated amino acids for 16 h, followed by treatment with LPS or LPS plus nigericin (LPS+Nig). The LDH release (**C**) and IL-1β secretion (**D**) were analyzed. N.D., not detectable. Experiments were repeated three times. Data are mean ± SD. *P < 0.05, **P < 0.01, ***P < 0.001, two-tailed *t*-test.

To test this, we selected a panel of neutral aliphatic amino acids identified in our analyses for functional screen (Supplementary Figure S1A). We also included 2-aminobutyric acid, which is structurally related to glycine but not detected in our metabolomic data, to serve as a comparator (Supplementary Figure S1A). We first evaluated the cytotoxicity of these amino acids in immortalized bone marrow-derived macrophages (iBMDMs) but did not observe significant cytotoxic effects at the tested concentrations (Supplementary Figure S1B). The cells were then pretreated with each amino acid prior to stimulation with LPS and nigericin, the canonical stimuli for NLRP3 inflammasome activation. Our results indicated that β-alanine significantly suppressed both morphological membrane blebbing and lactate dehydrogenase (LDH) release, a marker of membrane rupture, whereas glycine and alanine solely reduced LDH release (Figure 1C; Supplementary Figure S1C). The inhibitory effect of β-alanine on nigericin-induced pyroptosis was dose-dependent, with a half-maximal inhibitory concentration (IC_50_) of 2.17 mM (Supplementary Figure S1D). Furthermore, β-alanine, 2-aminobutyric acid, and isoleucine inhibited the release of mature IL-1β (Figure 1D). These findings suggest that β-alanine suppresses both membrane rupture and the release of the pro-inflammatory cytokine IL-1β during NLRP3 inflammasome-mediated pyroptosis.

### β-alanine specifically blocks NLRP3 inflammasome activation

To verify the inhibitory effect of β-alanine on NLRP3 inflammasome activation, we treated iBMDMs with β-alanine at two concentrations, 2 mM encompassing the experimentally determined IC_50_ of 2.17 mM for pyroptosis inhibition (Supplementary Figure S1D) and 4 mM reflecting the peak plasma concentration that is physiologically achievable in humans (Gonçalves et al., 2020). Pretreatment with β-alanine at these concentrations significantly reduced LDH release and mature IL-1β secretion (Figure 2A and B), accompanied by an obvious decrease in pyroptotic cell death as reflected by propidium iodide (PI) staining (Figure 2C; Supplementary Figure S2A). Immunoblotting analysis further validated the inhibitory effects of β-alanine on the cleavage of pro-caspase-1, pro-IL-1β, and GSDMD following stimulation with LPS and nigericin (Figure 2D). To determine whether β-alanine specifically modulates NLRP3-mediated inflammasome activation, we employed different stimuli that are known to activate either the NLRP3 inflammasome or other inflammasomes (Song et al., 2017). Our data revealed that β-alanine pretreatment significantly inhibited ATP- and MSU-induced NLRP3 inflammasome activation, exhibiting a concentration-dependent inhibitory effect (Figure 2E and F; Supplementary Figure S2B–G), but did not affect the activation of the absent in melanoma 2 (AIM2) inflammasome (Figure 2G; Supplementary Figure S3A and B), NLR family CARD domain containing 4 (NLRC4) inflammasome (Figure 2H; Supplementary Figure S3C and D), and non-canonical inflammasome (Figure 2I; Supplementary Figure S3E and F) induced by poly(dA:dT), *Salmonella Typhimurium*, and cytosolic LPS, respectively. Furthermore, we generated the *Nlrp3*-knockdown iBMDMs and verified the knockdown efficiency by quantitative polymerase chain reaction (qPCR) (Figure 2J). Our data showed that β-alanine pretreatment significantly inhibited nigericin-induced LDH release and IL-1β secretion in control cells (shNC), but this inhibition was completely abolished in *Nlrp3*-knockdown cells (Figure 2K and L). Together, these data establish β-alanine as a specific NLRP3 inflammasome inhibitor that effectively blocks NLRP3 inflammasome activation.

**Figure 2:**
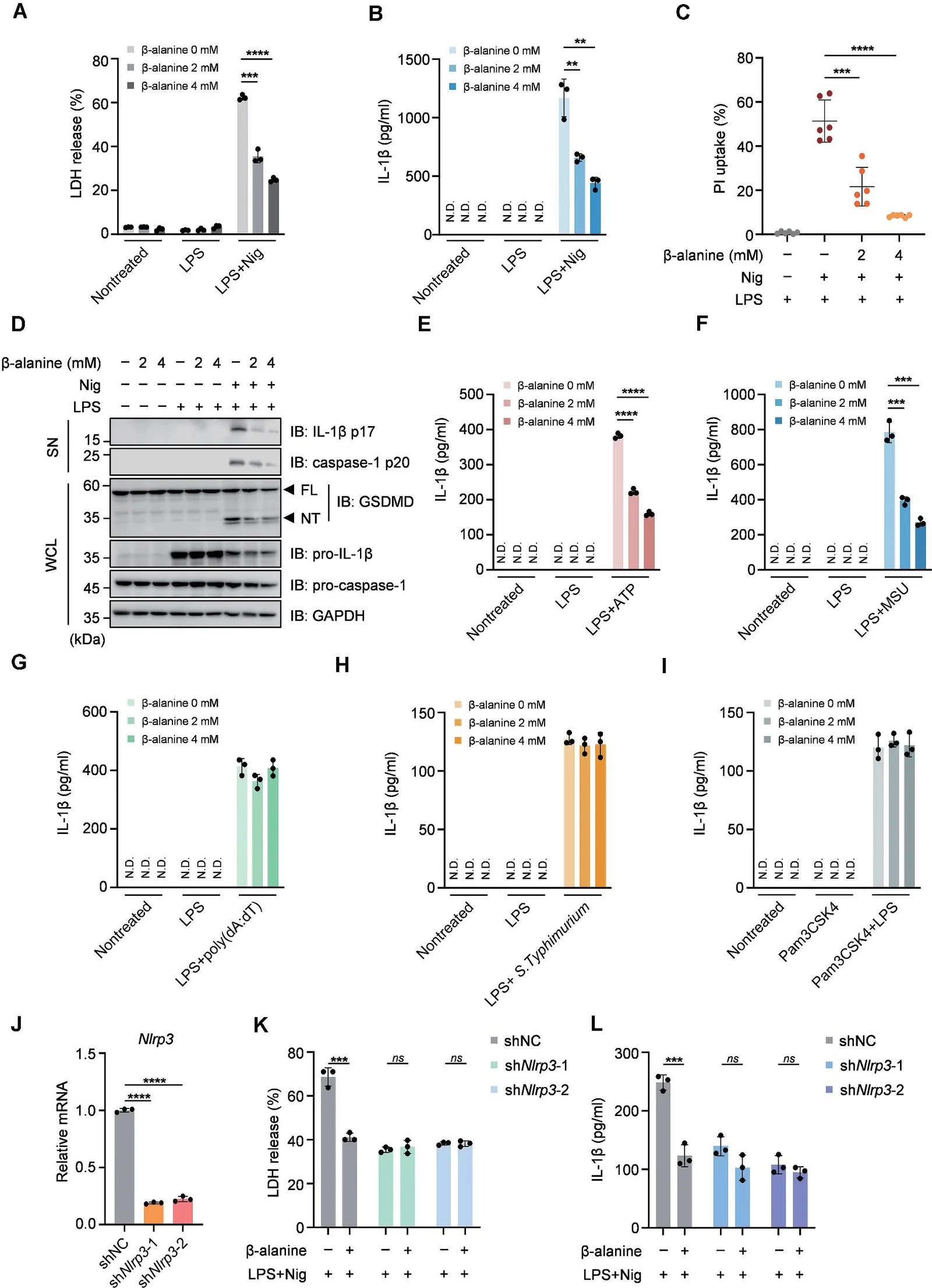
β-alanine specifically blocks NLRP3 inflammasome activation. (**A**–**D**) iBMDMs were pretreated with β-alanine at the indicated concentrations for 16 h. Cells were then treated with LPS or LPS+Nig or left untreated. The LDH release (**A**) and IL-1β secretion (**B**) were measured in the supernatants. (**C**) Cells were stained with PI and the percentage of PI-positive cells was quantified. (**D**) The whole-cell lysates (WCL) and supernatants (SN) were immunoblotted. FL, full length. NT, N terminal. (**E**–**H**) iBMDMs were pretreated with β-alanine at the indicated concentrations. The secretion of IL-1β in the supernatants from cells untreated or treated with LPS, LPS+ATP (**E**), LPS+MSU (**F**), LPS+poly(dA: dT) transfection (**G**), or LPS+*S. Typhimurium* (**H**) was analyzed by ELISA. (**I**) iBMDMs were pretreated with β-alanine at the indicated concentrations, followed by treatment with Pam3CSK4 or Pam3CSK4+LPS or left untreated. The secretion of IL-1β in the supernatants was analyzed. (**J**) qPCR verification of *Nlrp3* knockdown in iBMDMs transduced with control or sh*Nlrp3* lentivirus. (**K** and **L**) The *Nlrp3*-knockdown iBMDMs were pretreated with or without 4 mM β-alanine, followed by treatment with LPS+Nig. The LDH release (**K**) and IL-1β secretion (**L**) were measured in the supernatants. N.D., not detectable. Experiments were repeated three times. Data are presented as mean ± SD. *ns*, no significance. **P < 0.01, ***P < 0.001, ****P < 0.0001, two-tailed *t*-test.

### β-alanine disrupts NLRP3 inflammasome assembly

To elucidate the mechanism by which β-alanine inhibits NLRP3 inflammasome activation, we first assessed its impact on the expression of key inflammasome components during the priming phase. Immunoblotting analysis indicated that β-alanine did not affect the LPS-induced expression of NLRP3, pro-IL-1β, or other key components such as ASC (Supplementary Figure S3G). We next evaluated the assembly of inflammasome upstream of pro-caspase-1 cleavage during the activation phase. ASC speck formation and oligomerization serve as well-recognized indicators of inflammasome assembly (Martinon et al., 2002; Dick et al., 2016; Yu et al., 2023). Our results revealed that LPS plus nigericin stimulation induced significant formation of ASC speck in iBMDMs, which was markedly inhibited by β-alanine pretreatment (Figure 3A and B). Similarly, the formation of ASC dimers and oligomers following LPS and nigericin stimulation was suppressed in the presence of β-alanine treatment (Figure 3C). This disruption of inflammasome assembly may be attributed to the diminished recruitment of ASC to NLRP3, as analyzed by co-immunoprecipitation (co-IP) (Figure 3D). Notably, the interaction between NLRP3 and NEK7 was also reduced (Figure 3E), which is crucial for ASC recruitment and the subsequent speck formation (He et al., 2016). Thus, our data demonstrate that β-alanine disrupts the NEK7–NLRP3–ASC interactions, thereby impairing inflammasome assembly.

**Figure 3:**
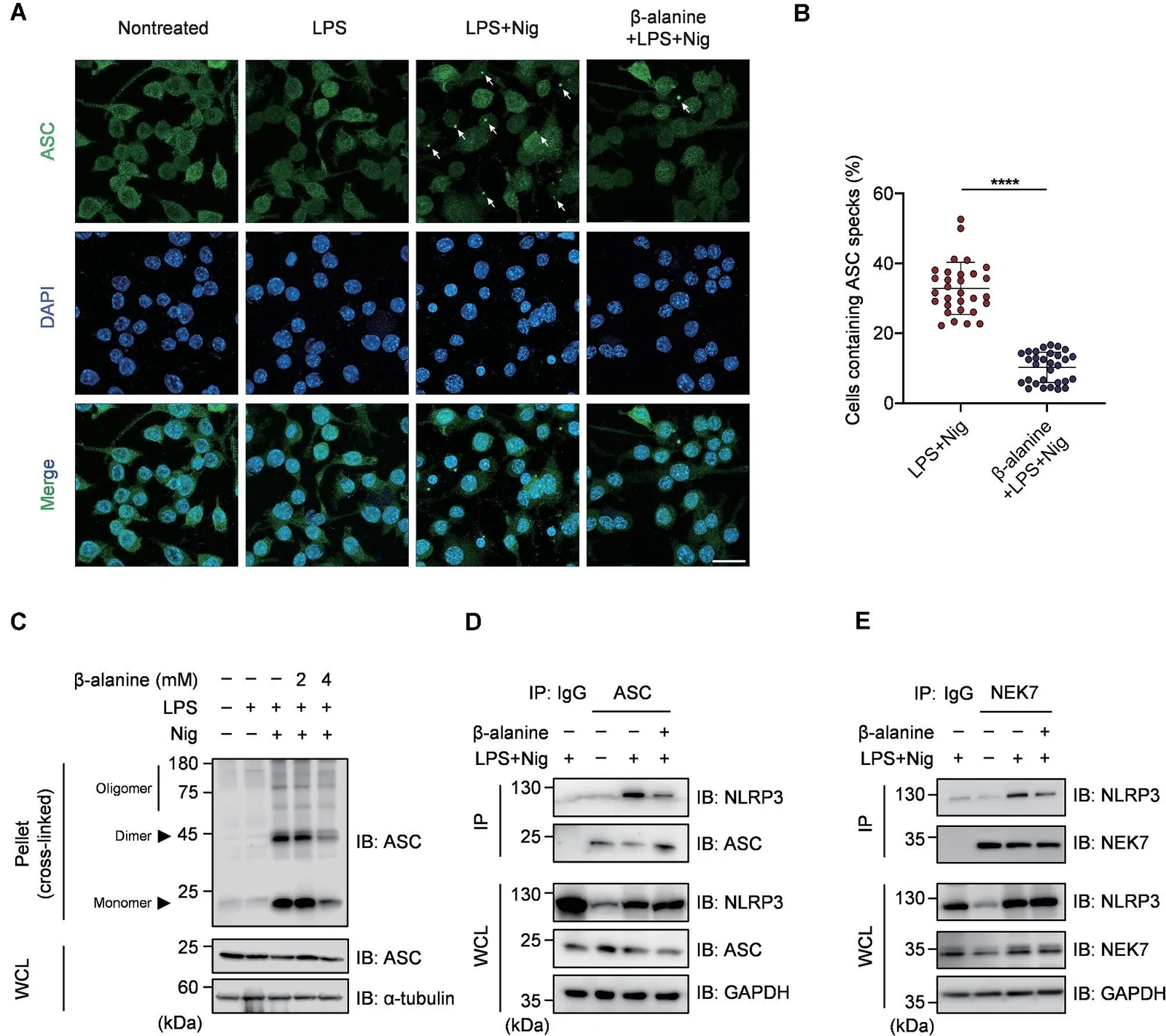
β-alanine disrupts NLRP3 inflammasome assembly. (**A**) Representative immunofluorescence images of ASC specks in iBMDMs treated as indicated. White arrows indicate ASC specks. DAPI, nuclear stain. Scale bar, 20 μm. (**B**) Quantification of the percentage of cells containing ASC specks in **A** (*n* = 30 fields). (**C**) Immunoblotting analysis of ASC oligomerization in LPS-primed iBMDMs stimulated with nigericin in the presence or absence of β-alanine at the indicated concentrations. (**D** and **E**) iBMDMs were primed with LPS for 3 h in the presence or absence of β-alanine, followed by stimulation with nigericin for 1 h. The interaction was analyzed by IP using anti-ASC antibody (**D**) and anti-NEK7 (**E**), followed by immunoblotting for NLRP3. IgG served as an IP control. Experiments were repeated three times. Data are mean ± SD. ****P < 0.0001, two-tailed *t*-test.

### β-alanine binds to NLRP3 and inhibits its trafficking from the TGN to the MTOC

Previous studies revealed that NLRP3 undergoes a conformational change, facilitating its trafficking from the TGN to the MTOC for subsequent recruitment of ASC (Chen and Chen, 2018; Magupalli et al., 2020; Fu and Wu, 2023). Building on these findings and our data indicating that β-alanine disrupts the interaction of NLRP3 with either NEK7 or ASC, we hypothesized that β-alanine may directly bind to NLRP3 and impede this essential translocation. Molecular docking simulations predicted that β-alanine exhibits the highest binding affinity for murine NLRP3 among all tested amino acids, forming key interactions with residues Gly225, Ile226, Gly227, Lys228, and Thr229 at the binding interface (Figure 4A; Supplementary Figure S4A). Consistent with this, pull-down assays demonstrated specific binding of β-alanine to NLRP3 (Figure 4B; Supplementary Figure S4B). The functional relevance of the predicted binding pocket was validated using a site-directed mutant, to which β-alanine binding was significantly impaired (Supplementary Figure S4C). We further quantified this interaction by isothermal titration calorimetry (ITC), showing a direct and high-affinity binding between β-alanine and NLRP3 with an equilibrium dissociation constant (*K*_d_) of 2.77 μM (Figure 4C). This finding was independently confirmed by surface plasmon resonance assays, yielding a comparable *K*_d_ value of 5.91 μM (Supplementary Figure S4D). These data collectively establish a specific and direct binding between β-alanine and NLRP3.

**Figure 4:**
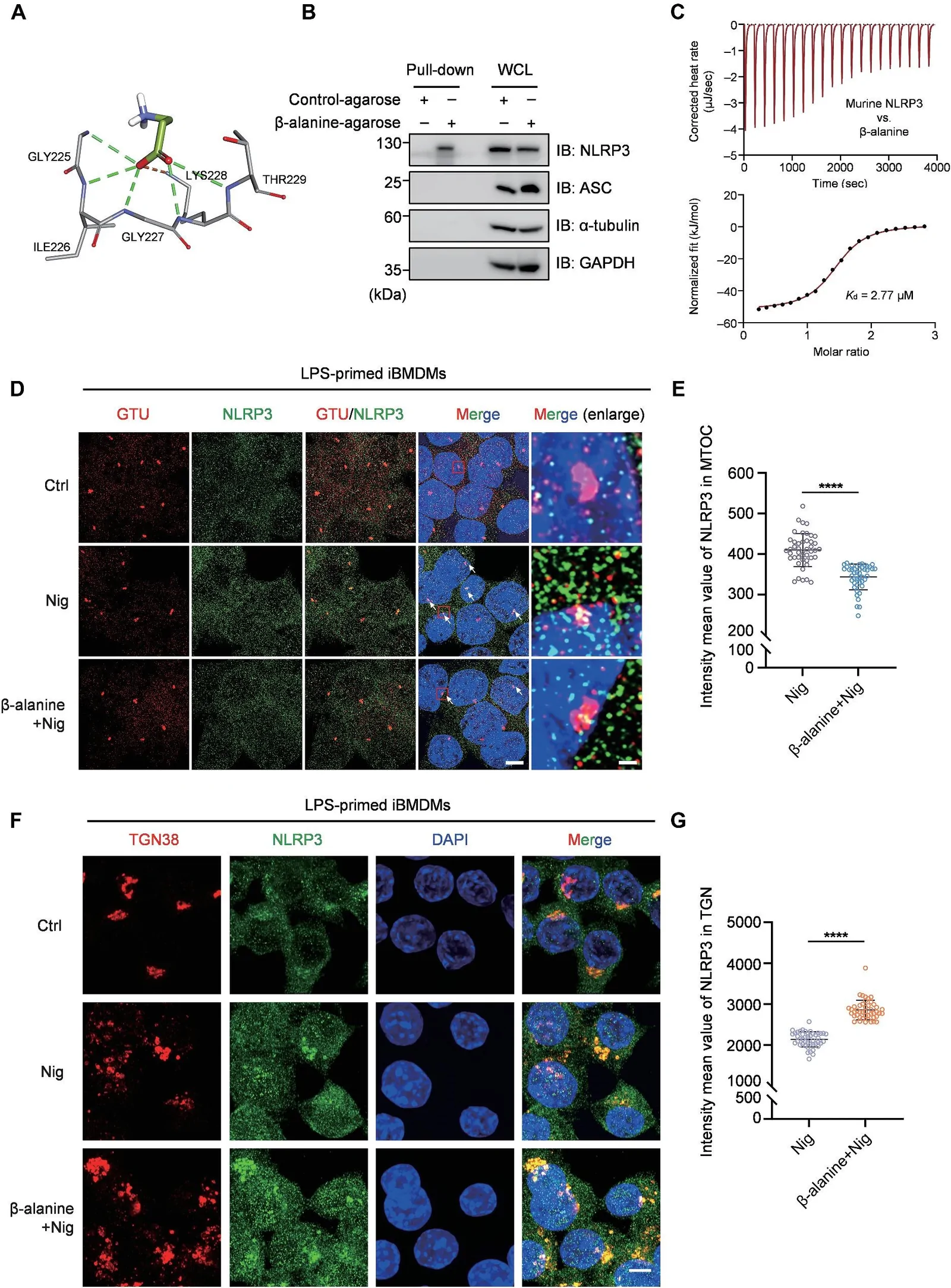
β-alanine binds to NLRP3 and inhibits its trafficking from the TGN to the MTOC. (**A**) Molecular docking simulations of β-alanine with murine NLRP3. The predicted binding site and potential covalent interactions between β-alanine and NLRP3 are shown. (**B**) The binding of β-alanine with NLRP3 was analyzed by pull-down assay in LPS-primed iBMDM lysates. (**C**) ITC assays were conducted to determine the binding affinity of β-alanine to NLRP3. (**D**–**G**) LPS-primed iBMDMs were stimulated with vehicle (Ctrl), nigericin, or nigericin and β-alanine. (**D** and **E**) Representative confocal images and quantification of the mean fluorescence intensity of NLRP3 co-localized with the MTOC marker γ-tubulin (GTU). Scale bar, 5 μm (left) or 0.5 μm (right). *n* = 45 microscopic fields. (**F** and **G**) Representative confocal images and quantification of the mean fluorescence intensity of NLRP3 co-localized with the TGN marker TGN38. Scale bar, 5 μm. *n* = 45 microscopic fields. DAPI, nuclear stain. Experiments were repeated three times. Data are mean ± SD. ****P < 0.0001, two-tailed *t*-test.

We next asked whether this binding influences the subcellular localization of NLRP3. Immunofluorescence analysis revealed that β-alanine pretreatment markedly reduced the aggregation of NLRP3 at the MTOC upon inflammasome activation (Figure 4D and E). Meanwhile, the average fluorescence intensity of NLRP3 co-localizing with the TGN was increased by β-alanine pretreatment (Figure 4F and G), indicating enhanced retention of NLRP3 at the TGN. These results collectively indicate that the binding of β-alanine to NLRP3 alters its subcellular redistribution, impeding translocation during inflammasome assembly.

### β-alanine mitigates LPS-induced systemic inflammation in vivo

Considering the pivotal role of the NLRP3 inflammasome in the pathogenesis of diverse inflammatory diseases, we next investigated the effect of β-alanine on mitigating NLRP3-associated inflammatory disorders *in vivo*. Mice were pretreated with normal drinking water or 1.2% (*w*/*v*) β-alanine in drinking water supplemented with an additional oral gavage every other day for 7 days, followed by challenge with inflammatory stimuli (Figure 5A). Our results showed that β-alanine administration significantly reduced the elevated plasma levels of IL-1β and IL-18 in response to LPS challenge (Figure 5B and C). Histological analysis with hematoxylin and eosin (HE) staining revealed a corresponding alleviation of LPS-induced tissue damage, including reduced hemorrhage in the lung and inflammatory cell infiltration and congestion in the liver (Figure 5D). Consistent with these findings, β-alanine-treated mice exhibited improved survival following a lethal LPS challenge (Figure 5E).

**Figure 5:**
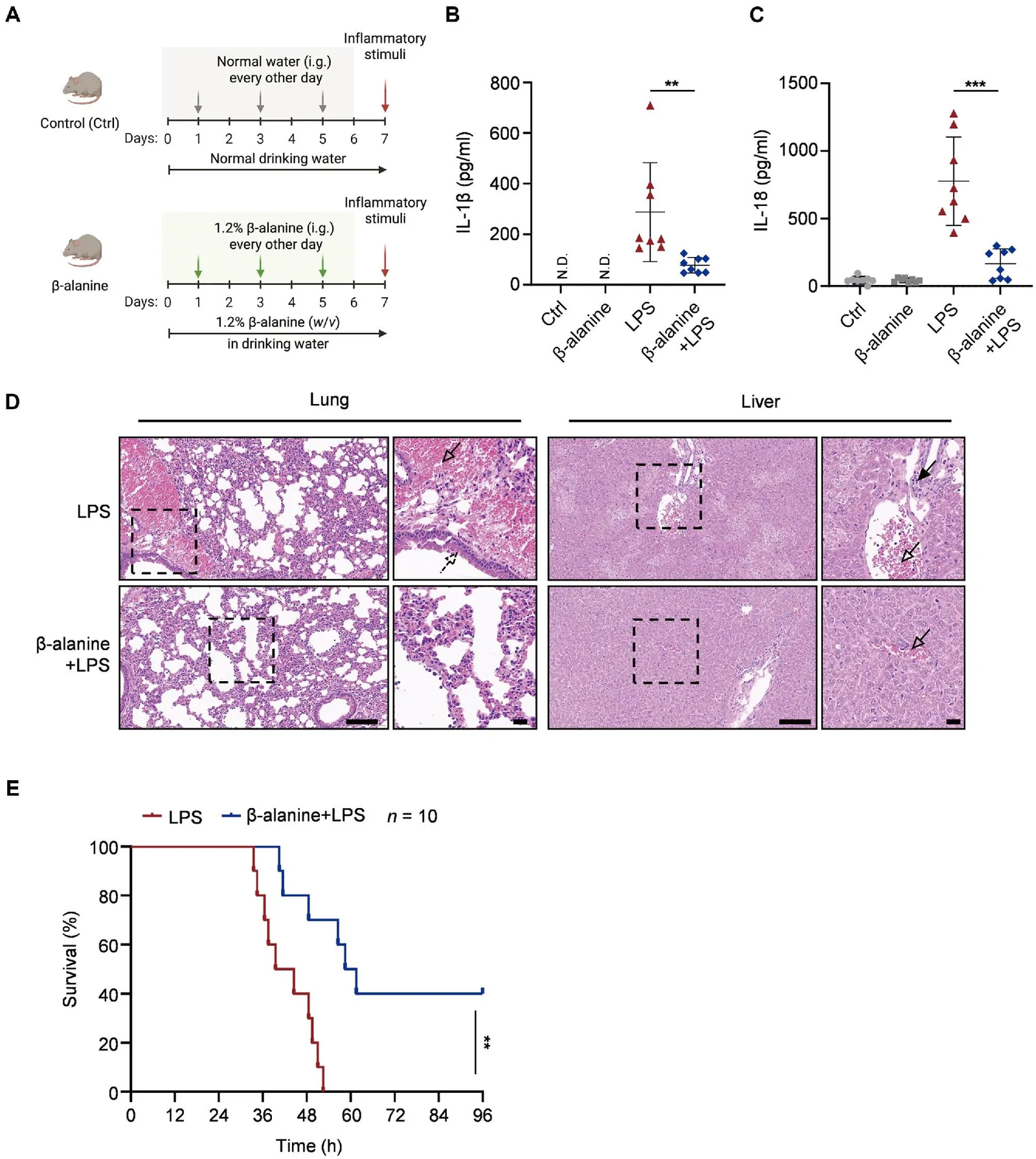
β-alanine mitigates LPS-induced systemic inflammation in *vivo*. (**A**) Experimental scheme for evaluating β-alanine in NLRP3-dependent inflammatory models. Mice received 1.2% (*w*/*v*) β-alanine in drinking water, with additional intragastric administration (200 μl of the same solution, i.g.) three times weekly. After 7 days of pretreatment, inflammatory stimuli were administered. (**B**–**D**) Mice (*n* = 8) pretreated with normal drinking water (Ctrl) or β-alanine-supplemented water were injected with PBS or LPS (20 mg/kg, *i.p*.). Plasma IL-1β (**B**) and IL-18 (**C**) were analyzed by ELISA after 12 h. N.D., not detectable. **P < 0.01, ***P < 0.001, two-tailed *t*-test. (**D**) Representative HE staining of lung and liver tissues from LPS-challenged mice. Scale bar, 50 μm. Solid line arrows with open arrowheads indicate hemorrhage and congestion. The dashed line arrow with an open arrowhead indicates eosinophilic material. The solid line arrow with a solid arrowhead indicates inflammatory cell infiltration. (**E**) Survival of mice (*n* = 10) pretreated with normal drinking water or 1.2% (*w*/*v*) β-alanine-supplemented water, followed by injection with 10 mg/kg LPS. **P < 0.01, two-sided log-rank (Mantel–Cox) test.

### β-alanine alleviates inflammation in MSU-induced acute gout arthritis model

We further assessed the anti-inflammatory efficacy of β-alanine in an acute gout arthritis model via intra-articular injection of MSU crystal. MSU-challenged mice developed ankle edema and the infiltration of inflammatory cells, both of which were attenuated by β-alanine treatment (Figure 6A). Additionally, MSU-induced IL-1β levels within the ankle joint also showed a marked decline after β-alanine treatment (Figure 6B). Longitudinal monitoring of ankle joint diameter revealed that β-alanine did not alter the initial swelling onset at 6 h post-MSU challenge but significantly accelerated the resolution of swelling thereafter (Figure 6C and D).

**Figure 6:**
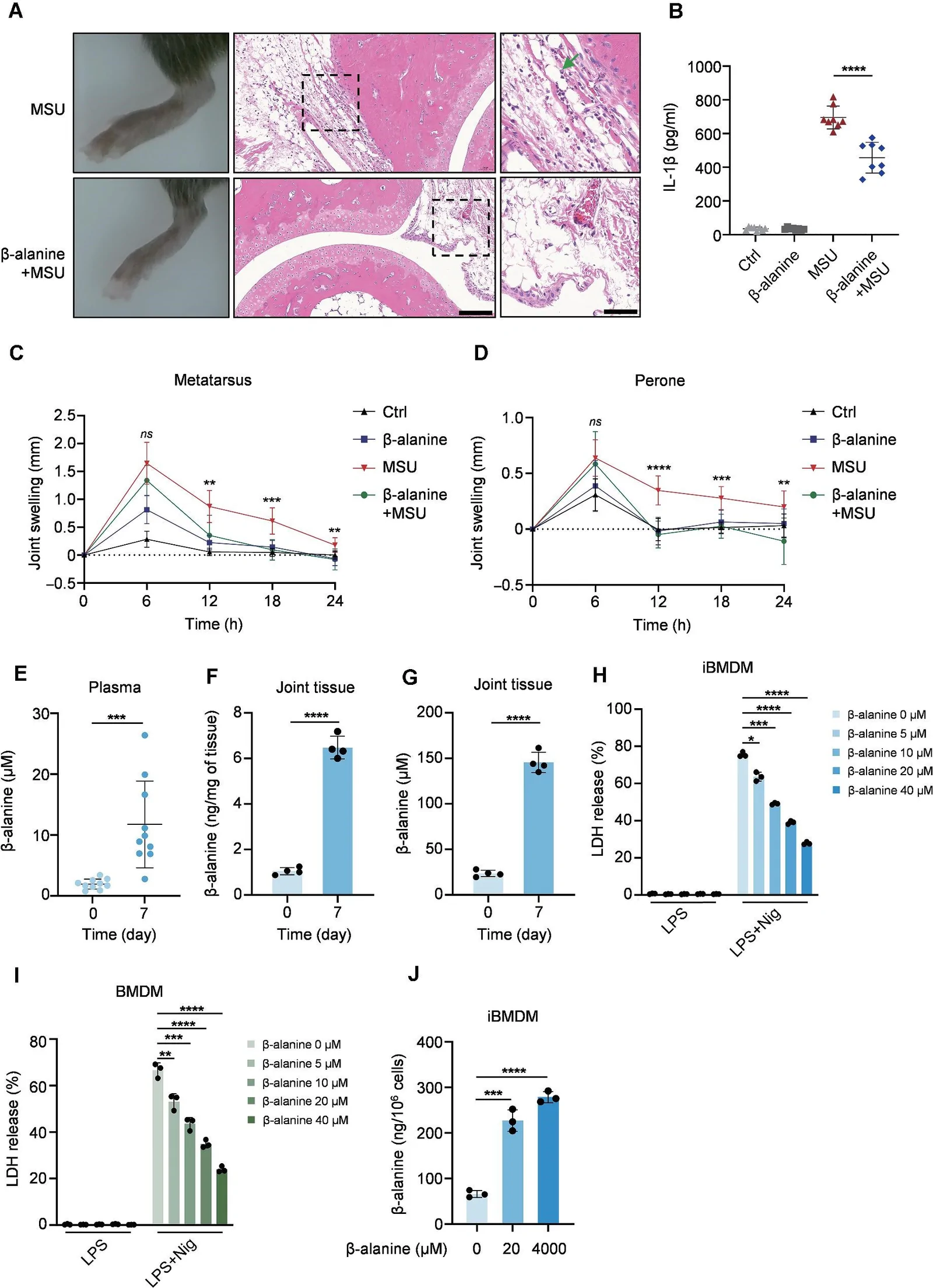
β-alanine alleviates inflammation in MSU-induced acute gout arthritis model. (**A**–**D**) Mice (*n* = 8) pretreated with normal drinking water (Ctrl) or 1.2% (*w*/*v*) β-alanine-supplemented water were injected with MSU crystals (0.8 mg in 20 μl PBS) or PBS into their joints. (**A**) Representative photomicrographs (left) and HE staining of joint tissues (middle and right) at 6 h post-injection are shown. Scale bar, 10 μm (middle) or 4 μm (right). The arrow indicates inflammatory cells infiltration. (**B**) IL-1β levels within ankle joint at 6 h post-injection were measured by ELISA (*n* = 8). ****P < 0.0001, two-tailed *t*-test. (**C** and **D**) Dynamic measurement of metatarsal (**C**) and fibular (**D**) joint swelling over 24 h post-MSU injection (*n* = 8). Data are mean ± SD. *ns*, no significance. **P < 0.01, ***P < 0.001, ****P < 0.0001, two-way ANOVA. (**E**–**G**) Mice received 1.2% (*w*/*v*) β-alanine in drinking water, supplemented with i.g. administration three times weekly. After 7 days, plasma β-alanine level of mice (**E**, *n* = 10) and β-alanine level in joint tissues (**F** and **G**) were determined by LC–MS. (**H** and **I**) iBMDMs (**H**) or BMDMs (**I**) were pretreated with β-alanine at the indicated concentrations for 7 days, followed by treatment with LPS or LPS+Nig. The IL-1β release was measured in the supernatants. (**J**) iBMDMs were treated with either 20 μM β-alanine for 7 days or 4 mM β-alanine for 16 h. Intracellular β-alanine levels in cell lysates were determined by LC–MS. Experiments were repeated three times. Data are mean ± SD. *P < 0.05, **P < 0.01, ***P < 0.001, ****P < 0.0001, two-tailed *t*-test.

Notably, this therapeutic effect was achieved under a chronic dosing regimen that resulted in a steady-state plasma β-alanine concentration of ∼12 μM as determined by liquid chromatography–mass spectrometry (LC–MS) (Figure 6E), which is considerably lower than the IC_50_ value of 2.17 mM observed in cellular assays. To address this apparent discrepancy and elucidate the basis of β-alanine’s *in vivo* efficacy, we analyzed the local distribution of β-alanine. Following the 7-day administration regimen, β-alanine levels in whole joint tissues were quantified and found to be enriched ∼5-fold relative to control mice (Figure 6F). This enrichment corresponded to an estimated concentration ranging from ∼135 μM to 161 μM after accounting for water content (Pethig and Kell, 1987; Turov et al., 2006), with a mean value of ∼146 μM (Figure 6G), demonstrating significant accumulation of β-alanine in local joint tissues. Based on these findings, we hypothesized that sustained, low-level exposure in combination with local tissue accumulation might underlie its pharmacological action. To test this, we modeled the chronic exposure *in vitro* by treating iBMDMs and primary BMDMs with low-dose β-alanine for 7 days. This treatment significantly suppressed NLRP3 inflammasome activation in a dose-dependent manner (Figure 6H and I; Supplementary Figure S5). Notably, ∼50% inhibition was achieved at ∼20 μM β-alanine, a concentration well below the high doses used in our initial short-term assays and within the range of our estimated levels in local tissues (Figure 6G). Supporting this effect, intracellular β-alanine was similarly enriched following both short-term high-dose and long-term low-dose treatments (Figure 6J), demonstrating efficient intracellular accumulation even at physiologically relevant low extracellular concentrations.

Collectively, these findings demonstrate that our β-alanine dosing regimen achieves functionally relevant concentrations within inflamed tissues and cells. This supports a mechanism whereby sustained intracellular and tissue accumulation of β-alanine enables effective suppression of NLRP3 inflammasome activation and consequent inflammatory responses *in vivo*.

## Discussion

Gout, a prevalent inflammatory arthritis characterized by MSU crystal deposition, remains a substantial global health challenge (Martinon et al., 2006; Dehlin et al., 2020). While current urate-lowering therapies mitigate hyperuricemia, the biochemical precursor to MSU crystal formation, they fail to resolve NLRP3 inflammasome activation triggered by persistent MSU crystals within joints (Jeong et al., 2021; Chen et al., 2024). This unresolved inflammation leads to chronic tissue damage and recurrent flares. In this study, through gout-associated metabolomic profiling, we identify β-alanine, an endogenous metabolite, as a novel and specific inhibitor of the NLRP3 inflammasome, highlighting the potential of targeting endogenous metabolic pathways for inflammatory control.

β-alanine, a non-proteogenic amino acid derived from endogenous synthesis or dietary sources (Trexler et al., 2015), is widely recognized for its function in elevating muscle carnosine levels to enhance exercise performance and fatigue resistance (Hoffman et al., 2015; Furst et al., 2018; Meftahi and Jahromi, 2023). The well-documented safety profile and established guidelines for its administration as a sports supplement offer a promising basis for investigating its potential as a therapeutic option for inflammatory conditions such as gout (Hoffman et al., 2015; Trexler et al., 2015; Furst et al., 2018; Wang et al., 2021; Meftahi and Jahromi, 2023). While our *in vivo* data demonstrate its efficacy in mitigating inflammation in both LPS-induced systemic inflammation and MSU-induced gout arthritis models, further examinations using human cell lines or patient-derived models are essential to validate its efficacy and safety in humans. The identification of β-alanine through the analysis of gout-associated metabolomics datasets further emphasizes the relevance of dysregulated amino acid metabolism in NLRP3-associated pathologies and the potential for exploiting endogenous pathways for therapeutic intervention. Thus, our finding adds β-alanine to a growing list of endogenous metabolites, such as itaconate and ketone bodies, which are recognized as key modulators of inflammasome activity (Youm et al., 2015; Hooftman et al., 2020; Shippy et al., 2020; Bambouskova et al., 2021), supporting the intricate link between cellular metabolism and innate immunity.

The potential therapeutic efficacy of β-alanine lies in its potent suppression of NLRP3 inflammasome activation and subsequent cytokine release. Mechanistically, we demonstrate that β-alanine binds directly to NLRP3, sequestering it within the TGN. This binding impedes NLRP3 trafficking and disrupts the NEK7–NLRP3–ASC interactions necessary for inflammasome complex formation at the MTOC, ultimately blocking pyroptosis and cytokine release. Notably, in the MSU-induced gout arthritis model, β-alanine treatment resulted in a distinct temporal profile. While it induced transient swelling when administered alone and did not attenuate MSU-induced swelling at the 6 h time point, it potently accelerated the resolution of swelling at later time points. We propose that this profile reflects the interplay between its pharmacokinetics and dual pharmacodynamic actions. The early swelling may be attributed to an NLRP3-independent vasodilatory effect of β-alanine (Cholewa et al., 2019), which could temporarily increase joint fluid volume without elevating IL-1β levels, thereby masking its concurrent anti-inflammatory activity against NLRP3. In contrast, the subsequent resolution of swelling aligns with sustained local tissue accumulation and intracellular enrichment of β-alanine. Under these conditions of chronic, low-level exposure, the inhibitory effect of β-alanine on NLRP3 inflammasome activation becomes dominant, leading to the suppressed IL-1β production and a pronounced acceleration of swelling resolution. Therefore, the temporal effects of β-alanine on gout arthritis may be explained by an initial vasodilatory response, while its therapeutic promotion of inflammation resolution is driven by sustained NLRP3 inhibition resulting from local tissue and intracellular accumulation. While our findings indicate that β-alanine binds to NLRP3 and inhibits NLRP3 translocation, the precise molecular details of this binding and its impact on NLRP3 conformational changes during trafficking need further investigation. Recent studies continue to highlight the Golgi apparatus as a critical hub for NLRP3 activation (Magupalli et al., 2020; Wang et al., 2023a; Zhang et al., 2023), providing a fertile ground for future research into the mechanisms of β-alanine.

In summary, our study uncovers a novel biological role for the endogenous metabolite β-alanine as an inhibitor of the NLRP3 inflammasome. This discovery not only expands the known biological functions of β-alanine, but also indicates that endogenous metabolites may represent a crucial, naturally evolved layer for regulating inflammatory responses, including NLRP3 activation. Given its favorable safety profile in humans for other indications, β-alanine represents a promising candidate worthy of further investigation as potential adjunctive therapy for gout and potentially other conditions driven by aberrant NLRP3 inflammasome activation.

## Materials and methods

### Cells

iBMDMs and primary BMDMs were cultured in Dulbecco's modified Eagle medium (DMEM) (MACGENE, CM10013), supplemented with 10% fetal bovine serum (EcCell, FSD500), 100 IU/ml penicillin, and 100 μg/ml streptomycin sulfate (MACGENE, CC004) at 37°C under a 5% CO_2_ atmosphere. iBMDMs were kindly provided by Prof. Feng Shao from the National Institute of Biological Sciences. Primary BMDMs were obtained by differentiating mouse bone marrow cells with rmM-CSF (25 ng/ml) for 7 days. All cells were confirmed mycoplasma-free by PCR.

### Mice

C57BL/6J mice (8-week-old) were purchased from Beijing Vital River Laboratory Animal Technology and maintained under specific pathogen-free conditions. All procedures were approved by the Institutional Animal Care and Use Committee (IACUC-NHLAC-2024-009).

### Immunoblotting

Cells were lysed in lysis buffer (20 mM Tris–HCl, pH 7.5, 0.5% NP-40, 250 mM NaCl, 3 mM EDTA, and 3 mM EGTA) containing 1 mM DTT, 1 mM PMSF, and protease inhibitor cocktail (Roche, 4906837001), and then mixed with 2× sodium dodecyl sulfate (SDS) loading buffer (WB-0081, Dingguo Biotechnology). For the detection of secreted IL-1β p17 and caspase-1 p20, supernatants from each group were collected and mixed with an equal volume of methanol, then combined with 200 μl chloroform. After centrifugation at 13000 rpm for 10 min at 4°C, the upper methanol layer was discarded without disturbing the protein interface. Proteins in the remaining liquid were precipitated with 600 μl methanol, followed by centrifugation at 15000 rpm for 10 min at 4°C. Pellets were air-dried and resuspended in 1× loading buffer. Proteins from both supernatants and cell lysates were denatured at 95°C and then separated by SDS–polyacrylamide gel electrophoresis. Anti-IL-1β antibody (R&D systems, AF-401-NA, 1:1000), anti-caspase-1 antibody (Adipogen, AG-20B-0042-C100, 1:2000), anti-GSDMD antibody (Abcam, ab209845, 1:2000), anti-NLRP3 antibody (Adipogen, AG-20B-0014-C100), and anti-GAPDH antibody (Proteintech, 10494-1-AP, 1:5000) were used. Proteins were visualized by enhanced chemiluminescence (Absin, abs920; Merck, WBKLS0500) according to the manufacturer’s instructions.

### PI staining

iBMDMs were seeded in 24-well plates and treated as indicated. Cells were incubated with 1 μg/ml PI (Sigma–Aldrich, P4170) in high-glucose DMEM. After 15 min of incubation, PI staining was visualized and images were acquired using a fluorescence microscope (Invitrogen, EVOS M5000).

### ELISA

For cell culture supernatants, iBMDMs were treated as indicated and the culture medium was collected. For ankle joint samples, tissues were harvested 24 h after intra-articular MSU injection, homogenized in M-PER reagent (Thermo Fisher Scientific, 78501). After centrifugation at 6000× *g* for 10 min, the supernatants were collected. For plasma samples, mouse blood samples were collected using anticoagulant tubes and centrifuged at 3000× *g* for 10 min at 4°C. Samples were then analyzed using mouse IL-1β and IL-18 ELISA Kit (Elabscience, E-EL-M0037, E-EL-M0730) according to the manufacturer’s instructions.

### Inflammasome activation

iBMDMs or BMDMs were seeded into 12-well plates at a density of 5 × 10^5^/well in the presence or absence of β-alanine (BBI, A606168-0100) at indicated concentration overnight. iBMDMs were primed with 200 ng/ml LPS (Invivogen, tlrl-eblps) for 3 h in Opti-MEM (ThermoFisher, 31985070), followed by stimulation as follows to activate the NLRP3 inflammasome: 7.5 μM nigericin (Invivogen, tlrl-nig) for 1 h, 5 mM ATP (Invivogen, tlrl-atp) for 0.5 h and 200 μg/ml MSU (Invivogen, tlrl-msu) for 4 h. Primary BMDMs were primed for 3 h with 200 ng/ml LPS, and then stimulated with 7.5 μM nigericin for 1 h. To activate the AIM2 inflammasome, 1 μg/ml poly(dA:dT) (Invivogen, tlrl-patn-1) were transfected into LPS-primed iBMDMs by Lipofectamine 2000 (ThermoFisher Scientific, 11668019) for 4 h. To activate the NLRC4 inflammasome, LPS-primed iBMDMs were stimulated with *S. Typhimurium* for 3 h (MOI: 10). To activate the non-canonical inflammasome, iBMDMs were primed with 1 μg/ml Pam3CSK4 (Invivogen, tlrl-pms) for 3 h, followed by transfection with 2 μg/ml LPS for 12 h.

### LDH Assay

The release of LDH was analyzed by CytoTox 96 Non-Radioactive Cytotoxicity Assay (Promega, G1780). In brief, 50 μl cell culture medium was mixed with an equal volume of CytoTox 96 Reagent in a 96-well flat clear bottom plate. Following dark incubation at room temperature for 15 min, 50 μl stop solution was added. The absorbance at 490 nm was recorded using SoftMax Pro 6.4 (SpectraMax i3).

### Immunofluorescence

iBMDMs were seeded on coverslips in 24-well plates and treated as indicated. Cells were fixed with 4% paraformaldehyde (PFA), permeabilized with 0.3% Triton X-100 and blocked in 5% bovine serum albumin. To visualize ASC specks, cells were incubated with rabbit anti-ASC antibody (Adipogen, AG-25B-0006-C100, 1:500) overnight at 4°C, followed by incubation with Alexa Fluor 488 goat anti-rabbit IgG antibody (Thermo Fisher, A-11008, 1:400) and DAPI (Beyotime, C1002, 1:1000). To visualize NLRP3 localization, cells were incubated with primary antibodies overnight at 4°C, including mouse anti-NLRP3, rabbit anti-gamma Tubulin (Abcam, ab179503, 1:200) and rabbit anti-TGN38 (Cell Signaling Technology, 65969S, 1:200). Alexa Fluor 488 goat anti-mouse IgG (Thermo Fisher, A-11001, 1:400), Alexa Fluor 546 goat anti-rabbit IgG (Thermo Fisher, A-11035, 1:400), and DAPI were subsequently used. Images were acquired using a ZEISS LSM 900 or LSM 980 confocal microscope.

### IP

iBMDMs were treated as indicated and then lysed on ice with lysis buffer containing protease inhibitors. Following centrifugation at 15000 rpm for 20 min at 4°C, the supernatants were collected and incubated with 5 μg indicated antibodies overnight at 4°C. Antibodies used are as follows: Anti-Normal Rabbit IgG antibody (Cell Signaling Technology, 2729S), anti-ASC antibody (Adipogen, AG-25B-0006-C100), and anti-NEK7 antibody (Abcam, ab133514). Proteins were precipitated by incubating with Protein G Sepharose beads (Cytiva, 17061801) at 4°C for 2 h, and analyzed by immunoblotting.

### Pull-down assay

Pull-down assays were performed using amino acid-conjugated beads. Briefly, cyanogen bromide-activated agarose beads (BBI, C500099) were incubated with 0.5 M amino acids in coupling buffer (0.1 M NaHCO_3_, pH 8.3, 0.5 M NaCl) overnight. After blocking with Tris–HCl buffer (pH 8.3) for 2 h at room temperature, beads were washed sequentially with sodium acetate buffer and Tris–HCl buffer and stored in 0.1 M Tris–HCl at 4°C.

For screening, lysates from LPS-primed iBMDMs were incubated with amino acid-conjugated beads. For binding comparison, HEK293T cells were transfected for 48 h with plasmids encoding either WT NLRP3 or a mutant harboring alanine substitutions (AAAAA) at residues Gly225–Thr229. Cell lysates were incubated with β-alanine-conjugated beads at 4°C for 10 h. Beads were then washed, and bound proteins were eluted and analyzed by immunoblotting.

### RNA isolation and qPCR

Total RNA was isolated using TRI reagent (Sigma, 93289). Subsequently, 500 ng of RNA was reverse-transcribed into cDNA using the PrimeScript RT reagent Kit (TaKaRa, DRR037A). qPCR was performed with PowerUp SYBR Green Master Mix (Applied Biosystems) on an ABI StepOnePlus system. The relative mRNA expression level of *Nlrp3* was normalized to *Hprt* and calculated using the comparative Ct method. The primer sequences used were as follows: *Nlrp3*-F, 5′-TGCGGATGGAATTTGGAACC-3′; *Nlrp3*-R, 5′-TGCCTCCTCTTCCAGCAAAT-3′; *Hprt*-F, 5′-TCAGTCAACGGGGGACATAAA-3′; *Hprt*-R, 5′-GGGGCTGTACTGCTTAACCAG-3′. All primers were synthesized by Tsingke Biotechnology.

### Generation of Nlrp3-knockdown iBMDMs

To generate *Nlrp3*-knockdown iBMDMs, two distinct shRNA sequences (sh1: GGATGAACGTGTTCCAGAAG; sh2: GGTCTCTTCTCAAGTCTAAC) targeting the 3′ UTR of *Nlrp3* mRNA were cloned into the lentiviral pLVX-Puro vector. Lentiviruses were produced by co-transfecting HEK293T cells with the lentiviral vectors, psPAX2, and pVSV-G at a ratio of 4:3:1. iBMDMs were then transduced with the resulting lentiviral particles. Stable knockdown cells were selected and maintained using 2 μg/ml puromycin.

### ASC oligomerization assay

ASC oligomerization was analyzed as described previously (Zangiabadi et al., 2022) with modifications. Briefly, cells were lysed in ice-cold buffer (20 mM HEPES, pH 7.4, 150 mM KCl, 0.5% Nonidet P-40, protease inhibitor cocktail). After centrifugation at 3400× *g* for 15 min, the pellets were washed with cold PBS and resuspended in PBS containing 2 mM disuccinimidyl suberate (MedChemExpress, HY-W019543) to cross-link oligomers at room temperature for 30 min. Following another centrifugation, the pellets were resuspended in loading buffer for immunoblotting analysis.

### In vivo models for NLRP3-related inflammatory disorders

For β-alanine treatment, mice received 1.2% β-alanine in drinking water (*w*/*v*) for 7 days and the water was replenished every 48 h. Additionally, 200 μl of the same β-alanine solution was administered every other day via oral gavage (Zong et al., 2024).

For LPS-induced systemic inflammation model, mice were injected intraperitoneally (*i.p*.) with 20 mg/kg LPS (Sigma–Aldrich, L2630) or PBS, and sacrificed after 12 h. The blood samples of mice were collected to measure cytokine plasma levels by ELISA. The livers and lungs of mice were collected for histological analysis. For survival analysis, mice were injected (*i.p.*) with 10 mg/kg LPS and monitored for survival over 96 h.

For MSU-induced acute gout arthritis model, 0.8 mg MSU (dissolved in 200 μl PBS) or PBS was injected into the ankle joints of mice. Joint swelling of metatarsus and perone was measured with a vernier caliper and photographed. Ankle joints of mice were collected for histological analysis and measurement of the pro-inflammatory cytokine levels via ELISA.

### HE staining

Tissues were immersed in 4% PFA for 24 h for fixation, and were then dehydrated and embedded in paraffin to enable histological sectioning. Paraffin blocks were sliced into sections with a thickness of 5 μm, followed by HE staining to evaluate inflammatory cell infiltration.

### Quantification of β-alanine by LC–MS

β-alanine was quantified by LC–MS. For cells, samples were lysed in pre-cooled 80% methanol, incubated at −80°C for 60 min, and centrifuged at 14000 rpm for 15 min. The supernatant was dried under vacuum. For tissues, samples were pulverized under liquid nitrogen and extracted with 80% methanol by low-temperature ultrasonication. After centrifugation, the supernatant was dried under vacuum. For plasma, mouse blood were collected in anticoagulant tubes and centrifuged at 3000 rpm for 20 min. The supernatant (plasma) was analyzed directly. All dried extracts were reconstituted in 60% acetonitrile before LC–MS analysis. For joint tissues, the measured β-alanine content was converted to an estimated tissue concentration (μM) based on a 50% water content assumption (Pethig and Kell, 1987; Turov et al., 2006).

### Data availability statement

The analysis presented in Figure 1A is based on data originally published by McCormick et al. (2024). For this study, numerical values were manually extracted from the supplementary materials of the original publication to identify differentially expressed amino acids.

The analysis presented in Figure 1B is based on data originally published by Renaudin et al. (2020). Numerical values were manually extracted from the supplementary materials of the original publication and subsequently re-analyzed via KEGG pathway enrichment for the current investigation.

### Statistical analysis

Statistical analyses were performed using GraphPad Prism. A standard two-tailed unpaired Student’s *t*-test was used for statistical analysis of two groups. Two-way analysis of variance (ANOVA) was used for statistical analysis between groups over time. Statistical analysis of survival curves was performed with a two-sided log-rank (Mantel–Cox) test. Data are presented as mean ± standard deviation (SD). *P* < 0.05 is considered as statistically significant.

## Supplementary Material

mjag004_Supplemental_File

## Notes

**Author contributions:** T.Z. and T.L. supervised the whole project. T.Z., T.L., and W.X. designed the experiments. T.Z., T.L., X.-Y.C., P.-P.Z., and Y. Yuan wrote the manuscript. P.-P.Z. performed the animo acid screening and immunofluorescent staining. X.-Y.C. performed the cell experiments and analyzed data using ZEISS LSM 900. Y. Yuan and L.-B.Y. performed mouse studies. Y.-Q.X. and M.-Y.F. performed the bioinformatics analysis. Y. Yu and W.X. helped with data analyses and discussions. T.Z. and T.L. acquired funding for the study.
