# Supplementary material for: β-alanine alleviates gout by inhibiting NLRP3 inflammasome activation
Source: J Mol Cell Biol. 2026 Apr 1;18:mjag004. doi: 10.1093/jmcb/mjag004 (PMC13684413; doi:10.1093/jmcb/mjag004)
Supplement: Supplementary file 1 [file mjag004_supplemental_file.docx]

**Supplementary Figure S1** **β-alanine suppresses characteristic membrane blebbing during pyroptosis.**

(**A**) Chemical structures of the indicated amino acids. (**B**) iBMDMs were treated with amino acids in (**A**) at indicated concentrations for 48 h. The cell viability was analyzed by MTS assay. (**C**) Representative microscopy images of LPS-primed iBMDMs stimulated with nigericin, in the presence or absence of 4 mM of the indicated amino acids or vehicle control (Ctrl). Scale bar, 50 μm. (**D**) LDH release assay of iBMDMs pretreated with a gradient concentration of β-alanine for 16 h, followed by stimulation with LPS alone or LPS plus nigericin. Nig, nigericin. Experiments were repeated three times. Data are mean ± s.d.

**Supplementary Figure S2** **β-alanine specifically suppresses NLRP3 inflammasome activation.** (**A**) LPS-primed iBMDMs were stimulated with vehicle (Ctrl), nigericin or nigericin and β-alanine at indicated concentrations. Representative images of PI staining were shown. Scale bar, 125 μm. (**B**-**D**) iBMDMs were pretreated with indicated concentrations of β-alanine, followed by treatment with LPS, LPS plus ATP or left untreated. The LDH release was measured in the supernatants (**B**). Representative images of PI staining were shown (**C**). Scale bar, 50 μm. Immunoblot analysis of WCL and SN was performed (**D**). (**E**-**G**) iBMDMs were pretreated with indicated concentrations of β-alanine, followed by treatment with LPS, LPS plus MSU or left untreated. The LDH release was measured in the supernatants (**E**). Representative images of PI staining were shown (**F**). Scale bar, 50 μm. The WCL and SN were immunoblotted (**G**). SN, supernatants. WCL, whole cell lysates. Nig, nigericin. MSU, monosodium urate. FL, full length. NT, N terminal. Experiments were repeated three times. Data are mean ± s.d. ****P* < 0.001, *****P* < 0.0001.

**Supplementary Figure S3** **β-alanine does not impair AIM2, NLRC4, or non-canonical inflammasome activation.** (**A** and **B**) iBMDMs were pretreated with indicated concentrations of β-alanine for 16 h, then left untreated or stimulated with LPS alone or LPS plus transfected poly(dA:dT). The WCL and SN were immunoblotted (**A**). The LDH release was measured in the supernatants (**B**). (**C** and **D**) iBMDMs were pretreated with indicated concentrations of β-alanine for 16 h. Cells were then treated with LPS, LPS plus *S. Typhimurium* (MOI = 10) or left untreated. The WCL and SN were immunoblotted (**C**). The LDH release was measured in the supernatants (**D**). (**E** and **F**) iBMDMs were pretreated with indicated concentrations of β-alanine for 16 h. Cells were then treated with Pam3CSK4, Pam3CSK4 plus LPS transfection or left untreated. The WCL and SN were immunoblotted (**E**). The LDH release was measured in the supernatants (**F**). (**G**) Immunoblotting analysis of iBMDMs left untreated or treated with LPS in the presence or absence of indicated concentrations of β-alanine. *S. Typhimurium, Salmonella Typhimurium.* SN, supernatants. WCL, whole cell lysates. FL, full length. NT, N terminal. Experiments were repeated three times. Data are mean ± s.d.

**Supplementary Figure S4** **Validation of the direct interaction between β-alanine and NLRP3.** (**A**) Molecular docking simulations predicting the binding affinities of the indicated amino acids to murine NLRP3. β-alanine showed the highest predicted affinity. (**B**) The binding of indicated amino acids to NLRP3 was analyzed by pull-down assay using lysates from LPS-primed iBMDM. (**C**) The binding of β-alanine to WT or mutant NLRP3 was analyzed by pull-down assay using lysates from HEK293T cells expressing WT or mutant NLRP3. Mut, refers to mutant NLRP3 carrying alanine substitutions (AAAAA) at the Gly225-Thr229 region. (**D**) The interaction between murine NLRP3 and β-alanine was analyzed by SPR, with a calculated *K*_d_ of 5.91 μM. *K*_d_, equilibrium dissociation constant. WCL, whole cell lysates.

**Supplementary Figure S5 Long-term, low-dose β-alanine treatment suppresses NLRP3 inflammasome activation.** (**A**) IL-1β release from iBMDMs pretreated with indicated concentrations of β-alanine for 7 days, followed by stimulation with LPS or LPS plus nigericin. (**B**) IL-1β release from BMDMs pretreated with indicated concentrations of β-alanine for 7 days, followed by stimulation with LPS or LPS plus nigericin. Nig, nigericin. Experiments were repeated three times. Data are mean ± s.d. N.D., not detectable. **P* < 0.05, ***P* < 0.01, ****P* < 0.001, *****P* < 0.0001, two-tailed *t* test.
